# Stimulus Control Procedure for Reducing Vocal Stereotypies in an Autistic Child

**DOI:** 10.3390/children8121107

**Published:** 2021-12-01

**Authors:** Marco Esposito, Laura Pignotti, Federica Mondani, Martina D’Errico, Orlando Ricciardi, Paolo Mirizzi, Monica Mazza, Marco Valenti

**Affiliations:** 1 Department of Applied Clinical Sciences and Biotechnology, University of L’Aquila, 67100 L’Aquila, Italy; monica.mazza@univaq.it; 2 Department of Psychology, University of Rome La Sapienza, 00185 Roma, Italy; la.pignotti@gmail.com; 3 Autism Research and Treatment Centre, Una Breccia nel Muro, 00168 Roma, Italy; federica.mondani@unabreccianelmuro.org (F.M.); orlandoricciardi1989@gmail.com (O.R.); 4 Modern Cultures and Literatures, University of Rome La Sapienza, 00185 Roma, Italy; derrico.1797479@studenti.uniroma1.it; 5 Department of Psychology, University of Bari, 70121 Bari, Italy; paolomirizzi@yahoo.it; 6 Regional Centre for Autism, Abruzzo Region Health System, 67100 L’Aquila, Italy; marco.valenti@univaq.it

**Keywords:** vocal stereotypies, autism spectrum disorder, automatic reinforcement, stimulus control, changing criterion design

## Abstract

Stereotyped vocal behavior exhibited by a seven-year-old child diagnosed with autism spectrum disorder and maintained by automatic reinforcement was placed under stimulus control through discrimination training. The training consisted of matching a green card (SD) with free access to vocal stereotypy and a red card (SD-absent) with interruption of stereotypy and vocal redirection. At the same time, appropriate behaviors were reinforced. After discrimination training, the child rarely engaged in vocal stereotypy in the red card condition and, to a greater extent, in the green card condition, demonstrating the ability to discriminate between the two different situations. After the training, the intervention began. Once they reached the latency criterion in the red stimulus condition, the child could have free access to vocal stereotypy (green card condition). The latency criterion for engaging in stereotypy was gradually increased during the red card condition and progressively decreased during the green card condition. The intervention follows a changing criterion design. This study indicates that stimulus discrimination training is a useful intervention to reduce vocal stereotypy in an autistic child.

## 1. Introduction

Children diagnosed with autism spectrum disorders (ASD) are characterized by social communication deficit and a tendency to engage in a pattern of restricted and repetitive behavior [1]. Moreover, autistic children can present impairments in a large variety of developmental abilities, from social–cognitive to behavioral challenges. Although cases of autism have been increasing, currently effective educational interventions for these children are widely diffused, and progress has been demonstrated in intellectual, language, behavior, social, and adaptive skills [2]. Stereotypies are included in core symptoms and they are defined as topographically invariant and repetitious behaviors [3], representing a diagnostic feature of autism spectrum disorder (ASD) according to the current diagnostic criteria of the Diagnostic and Statistical Manual of Mental Disorders. Vocalizing, echolalia, and a-contextual speech are examples of stereotypies maintained by automatic positive reinforcement [3,4]. Stereotypies can become a barrier to learning if their reinforcing value exceeds other stimuli in the environment, and the behavior absorbs too many daily activities of children. Indeed, stereotyped behaviors may compete with other activities and develop control of the stimulus and instruction interfering with learning [5]. In addition, stereotypies can be socially stigmatizing and limit opportunities for socialization and learning in daily life, such as engagement in leisure, vocational, self-care, and may interfere with skill acquisition in academic settings [3,6,7,8]. Regarding academic settings, Cook and Rapp [9] guide teachers and clinicians through different considerations prior to the implementation of behavior interventions. In sum, the extent to which direct treatment of vocal stereotypy is required for academic tasks varies on an individual basis; essentially, an assessment concerning some conditions including the impact of standard teaching on stereotypies or access of music and others aspects could evoke more or less the stereotypies in children. The mentioned model can also guide practitioners in selecting the least intrusive and most efficient intervention during the academic curriculum. For example, an individual may require multiple preferred items to compete with stereotypy during conditions with low ambient stimulation and adult interaction, but may require either a different or no intervention during academic instruction since only instructions and praises can limit challenging behaviors. At same time, consider the individual without his environment as school context can shed light only on the part of the current issue. Also, treating stereotypies could decrease the stigma that others perceive when children with ASD displays the behavior, impeding social interactions. A recent study [10] has administered a questionnaire to college students after having watched a child engaging in the motor and vocal stereotypy evaluating their perception. Results indicated that observers negatively rated the child when he displayed motor stereotypy, the additional vocal stereotypy yielded more negative judgements than motor stereotypy alone. Stereotyped behaviors exhibit several topographies, including echolalia [11], repetitions of meaningless sounds [12], instances of non-contextual or non-functional speech [13]. Echolalia behavior may be immediate or delayed. Fay [14] defined immediate echolalia as “meaningless repetition of a word or word group just spoken by another person” (p. 39). Differently, delayed echolalia requires longer durations, including repeats of expressions heard even several days earlier. There seems to be a lack of understanding of the sound being repeated and of communicative intent in both cases.

### 1.1. Function Analysis of Stereotypies

Several studies have reported that reinforcement contingencies could maintain vocal and motor stereotypy behavior for access to attention [15,16,17,18] and by reinforcement contingencies for access to the tangible [15]. However, according to Rapp and Vollmer [3], more than 90% of the published functional analyses of stereotypies show that they are maintained by automatic positive reinforcement, a stimulus produced independently of the social environment. Results indicate that the repetitive behaviors (motor and vocal) observed on different individuals with various developmental disabilities are reinforced by the stimulation that was directly generated by the behavior [19], on the other hand, it is maintained by the sensory consequences it produces. Additionally, although repetitive vocalizations may be maintained by social consequences, a recent review [20] focused report behaviors maintained by nonsocial consequences since: (a) stereotypy generally persists in the absence of social reinforcement; (b) interventions for socially reinforced vocalizations considerably differ and would require a separate review; and (c) repetitive vocalizations maintained by social consequences would be more accurately labeled using the verbal operants described and defined by Skinner. Therefore, the current study uses the term vocal stereotypy to refer to any repetitive sounds or words produced by an individual’s vocal apparatus that are maintained by nonsocial reinforcement.

### 1.2. Behavioral Interventions

Vocal and motor stereotypies maintained by automatic reinforcement are difficult to treat due to the challenging control of access to and retention of the sensory consequence it produces [21]. Some studies have used mild punishment to treat automatic reinforcement in vocal stereotypies in individuals diagnosed with autism. For example, Ahearn, Clark, MacDonald and Chung [22] found that target response interruption and redirection to appropriate vocalizations (RIRD; response interruption and redirection) positively reduced vocal stereotypy in at least four interventions with autistic children. The findings were later confirmed by Athens and Vollmer [23] and Cassella, Sidener, Sidener and Progar [24]. Interventions based on RIRD [25] involve interrupting vocal or motor stereotypy and replacing it with response exercises to simple questions concerning social rules or how to request an object [22]. This procedure conforms to ethical requirements that behavior analysts do not use aversive methods or punishment procedures until all reinforcement options have been exhausted [26]. In RIRD, the response blocking of the stereotypies is associated with the social reinforcement provided by therapist in contingency of the appropriate vocal responses of the child [24]. Rapp, Patel, Ghezzi, O’Flaherty, and Titterington [27] have highlighted how antecedent—and consequence—based interventions reduce vocal stereotypy in children with ASD [12,13,22,28]. Research has highlighted how effective discrimination training is on problem behavior maintained by automatic reinforcement. Discrimination training is a procedure that reinforces a target behavior in the presence of certain antecedent stimuli, indicates that the reinforcer will not be available in that contingency, and prevents reinforcement of the same target behavior in the presence of other antecedent stimuli [29]. The outcome of such training involves the subject producing appropriate responses in the presence of predictive reinforcing stimuli and appropriate behaviors (the non-emission of stereotyped behavior) in the presence of predictive extinction or punishment stimuli. This stimulus control procedure has been used in some research to teach adaptive responses to individuals diagnosed with autism, including communication, social, academic, vocational, and self-care skills [30,31]. Several recent studies document the effects of decreasing maladaptive behavior by making use of discrimination training [27,32,33,34,35]. Rapp et al. [27] and O’Connor, Prieto, Hoffmann, DeQuinzio, and Taylor [36], referring to previous studies, confirmed and demonstrated that environmental stimuli can exert an inhibitory control on behavior automatically reinforced after discrimination training. Specifically, they combined a red card using a verbal reprimand (positive punishment) or toys removal (negative punishment) to interrupt vocal stereotypy. In contrast, no programmed consequences were applied when the occurrence of stereotypy was paired with a green card. Their research suggests that stereotypy passed under the card’s control occurring less in the presence of the red card and more when paired with the green card. The basic principle of this procedure is that the green card stimulus assumes the function of conditional positive reinforcement when applied contingently to the absence of stereotypy in the red card condition [32]. During the intervention, when the subject met the criterion in the red card condition, he had access to the green card condition (access to stereotypy). The criterion consisted in the non-emission of stereotypy for a progressively longer time. At the same time, the duration of access to the green stimulus was systematically decreased. The red and green stimuli were scaled down from posters to 10 cm colored cards, and stimulus control was generalized to the participant’s classroom and community environment (public library). Results were discussed in terms of discrimination training as a useful intervention for reducing motor and vocal stereotypy. Also, a more recent study [37] reported the effects of a stimulus control procedure involving contingent reprimands on stereotypies of five children with ASD. A brief functional analysis indicated that motor and vocal stereotypy persisted in the absence of social consequences. Subsequently, results indicated that contingent verbal reprimands decreased the targeted stereotypy of the children. The present study describes and evaluates the effectiveness of the application of discrimination training using a RIRD procedure. The main objective was to reduce stereotyped vocal behaviors and implement contextually appropriate alternative behaviors during solitary play in a seven-year-old child diagnosed with autism.

## 2. Method

### 2.1. Participant

The participant, Robert, was a seven-year-old boy diagnosed with ASD according to the criteria of the DSM–5 [1]. The child attended the first year of primary school. Robert followed an applied behavior analysis (ABA) treatment at a center and home for 8-h a week (divided in 4 h at clinical center and 4 h at home). Please, note that we refer to the child using disability-first language against the use of first-person one, since the disability is part of a person’s identity [38]. Robert engaged in vocal stereotypy for much of his play time and at school. His parents and school teachers reported that this behavior interfered with his learning distressing his classmates. At school, the main difficulties of the child were reported in sustained attention to academic activities, including play time with peers. The family required to clinical staff a specific intervention for the reduction of the vocal stereotypies and provided informed consent in order to participate to the training. The child profile concerning abilities was assessed using the revised Assessment of Basic Language and Learning Skills [39]. Robert was able to give attention to an adult and materials. He described a short story with flashcards, simple comments with respect to an object or activities, and answered questions regarding objects, details, function, categories, and personal characteristics. Robert utilized adjectives and some verbs in phrases. Likewise, his request level was formed by common verbs, nouns, and adjectives (generally, three or four words every request). At the time of the current study, he was learning the past simple tense to describe events along with reading-writing letters and syllables. Concerning social interaction, Robert preferred to play alone; some social initiatives were supported by therapists. Nevertheless, the child played in turn with other peers but he didn’t engage in symbolic plays. Finally, preschooler autonomies and imitations were preserved, and no physical impediments were observed. Concerning his challenging behaviours about stereotypies, Robert frequently repeated entire strings of cartoons that he had previously watched on television or YouTube channels (e.g., “Like a little bird from my nest I will now fly”). The behavior was defined as delayed echolalia, since it occurred hours or days after the first contact with the stimulus. In order to understand the recurrent function of the above-mentioned behavior, we applied a descriptive functional assessment (Antecedent-Behavior-Consequence, ABC) which suggested us that the vocal stereotypy of Robert occurred in different environments, especially while he was playing alone in his room. The behavior appeared to be maintained by automatic reinforcement. Although the clinical staff well-identified the function of the behavior, we suggest the use of ABC narrative recordings and open-ended functional assessment interviews along with traditional functional analysis (FA), since they have reported limited empirical support (47% and 71% of cases, respectively, matched with FA) concerning hypotheses on the functions of challenging behavior [40].

### 2.2. Setting and Materials

Experimental sessions were held in the clinical room at the center and at home of the child with two therapists, two sessions a week. The rooms were furnished with a desk and chairs, a large rug, and various toys and books. Throughout the discrimination training, the child alternated play sessions either at the desk or on the carpet. The intervention started at home while the last three sessions were held at the clinical center. It was not possible to conduct the intervention at school due to restrictions related to the COVID-19 emergency. The discrimination training consisted of presenting two discriminative stimuli during the child played on the carpet or table with high preference available toys. The plays most selected were the marble race truck and a book with pictures and written words. Two 10 cm × 10 cm red and green cards were used as stimuli for discrimination training, such as in the study of Rapp et al. [27] and O’Connor et al. [36]. The green card was faded by resizing to 5 cm × 5 cm during the training until its gradual removal with no card condition, whereas a red elastic bracelet replaced the red card for a major portability (this arrangement could guarantee a more efficacy training during transitions in different places).

### 2.3. Data Collection and Dependent Variable

For all conditions of the experiment, the dependent variable was the occurrence of stereotyped vocal behavior defined as vocalizing: (a) non-communicative and not appropriate to the context (e.g., repeating movie phrases or words while playing with the track) or (b) repetitive (more than three repetitions of phrases or words within 15 s). Appropriate behavior was defined as playing without vocal stereotypy. Therefore, the dependent variable was the latency between the verbal instruction, “You can play now”, given by the therapist and the first exhibition of the vocal stereotypy (the therapist measured the latency time with a digital stopwatch); this data collection based on latency was applied only during the baseline and intervention phase. Conversely, during discrimination training, stereotypy was scored on a partial interval time recording (PIR) into a 5-min session divided into twenty 15-s intervals when the child remained alone. Consequently, the intervals were scored as positive if the participant engaged in stereotypy at any point during the 15-s. The dependent measure in the discrimination phase was, hence, the percentage of 15-s intervals engaged in stereotypy during playing. On the contrary, when the participant did not engage in stereotypy for the entirety of the 15-s interval (whole interval), it was scored as negative. The measurement was converted to the percentage of time when the child was engaged in stereotyped vocal behavior (number of positive intervals divided number of totals intervals × 100). Inter-observer agreement (IOA) was calculated by two therapists only for discrimination training by dividing the number of agreements by the number of agreements plus disagreements and multiplying by 100. Treatment integrity data were collected for 30% of sessions at a mean score of 100% accuracy.

### 2.4. Experimental Design

Three conditions were implemented in this study. Firstly, a baseline assessment was conducted, followed by discrimination training and an intervention phase. The purpose of the discrimination training was to control vocal stereotypy by matching programmed consequences with red and green stimuli. At the same time, the child played alone with building blocks, marble tracks, or books of his choice. During the intervention phase, the experimental design used was the changing-criterion design to evaluate the effectiveness of the intervention on increasing the latency time in which the child engages in vocal stereotypy.

### 2.5. Procedure: Baseline, Discrimination Training and Intervention

In the baseline condition, Robert was in his room, sitting or standing and having free access to various familiar plays. The child was alone while the therapists could observe him from another room via the video camera. In baseline condition, the stereotyped vocal behavior was not interrupted, and no reinforcement or suggestions were provided during appropriate play. Similarly, red and green cards were not present. The eight sessions lasted one minute and successively the discrimination training started. In order to bring the behavior of the child under the stimulus control, discriminating the color of the cards, we have delivered a positive punishment in the presence of the red color (reducing stereotypies) and positive reinforcement in the presence of the green color (free access for stereotypies). In the red card condition Robert was sitting at the table and was allowed to access his toys. The therapist was behind the child and pointed to him the red card saying “Red card, you play without talking”. As a result, when the child emitted vocal stereotypies, the contingency included a mild reprimand (“Red card, you play without talking”), the interruption of the game, and vocal redirection (e.g., “How many balls are missing?”, “I see a dog with...”). Therefore, when the behavior was appropriate (contextual commentary on the play activities), the contingency included reinforcement with tangibles (e.g., access to the game and more pieces of a toy) and a praise with encouragement (descriptive reinforcement such as “Good, you are playing well, yes the balls are yellow”). The green card condition followed the red card condition. In this condition, the child was seated at the small table and had free access to his games, while on the table was a green card that the therapists pointed to by saying, “Green card, you can play”, leaving him alone in the room. There were no contingencies in this condition, so Robert could continue to play emitting stereotypies. The discrimination training was divided into five phases (every phase comprised a different number of the sessions lasted five minutes), each representing a different schedule of reinforcement. Reinforcements (tangible and praise) were identified through a preference assessment, and they were delivered based on a variable interval (IV) reinforcement schedule, contingently the appropriate behavior. Once the child reached zero rates of stereotypy, the reinforcement schedule was thinned. Reinforcements were provided on average every 15 s (I and II phase), every 20 s (III phase), and every 25 s (IV phase). In the second phase, the red bracelet placed on the child’s wrist replacing the red card. The fifth phase did not include any reinforcement or prompt.

The intervention began after the discrimination training. The intervention included 32 sessions, each lasted five minutes. A changing criterion design was applied to assess the effectiveness of the green stimulus (or simply red card removal) as an access to automatic reinforcer, in order to increase the latency to engage in stereotypy during red conditions (since green stimulus occurred after the removal of red one, the free access to stereotypy could become a motivation for Robert engaging less with vocal stereotypies in order to have the access to automatic behaviour). Hence, reaching criterion in one condition, the child was required not to emit stereotypy for a longer time to access the automatic reinforcer. Anyhow, the sessions took place in his bedroom and in living room/kitchen. The child was sitting at the table or on the carpet and had access to his toys. The child played alone while his mother was busy with daily activities. The last three sessions were held in the centre with different therapists. During the intervention, the red card condition was called red bracelet and the green card condition (from session 35 onwards) no card (gradually we faded the green stimulus). During the red condition, the therapist put on child’s wrist the red bracelet saying, “now you can play”. After the instruction, he started the stopwatch monitoring the child from a video connected to the video camera. Contrary to discrimination training, the therapist did not give instructions such as “Red card, you play without talking” and did not use any prompts or additional reinforcement to the contextual comments. When Robert did not engage in the stereotyped vocalizations, he was allowed access to the green card/no card condition for the residual time of the 5-min session (since the sessions lasted 5 min, when the child reached the predefined latency criterion starting by 30 to 300 s during the sessions as showed in Table 1, if he respected the latency criterion could access or not to stereotypy consuming the residual time of each single session). On the other hand, if he showed stereotypies before the end of the predefined criterion, Robert wouldn’t have access to the green card condition, and the session ended. During the green card/no card condition, the child did not wear the red bracelet and could emit the target behaviour without intervention. In the red bracelet condition, the latency criterion in order to access to automatic reinforcement (access to the green card/no card condition) was progressively greater during each session:

### 2.6. Generalization of the Intervention

Generalization was assessed conducting the last three sessions of the intervention in the clinical room with different toys and activities, likewise different operators, and interacting with a peer. Robert was sitting at the table with another child sharing a construction game (sessions 38–39). The child was wearing only the red bracelet. However, he was not reminded of the condition. After having reached the predefined criterion (5 min), the bracelet placed on the wrist was removed, leaving free access to the stereotypies. During session 40, Robert was on the carpet alone, wearing the red bracelet. He was watching a book labelling pictures and reading the written words. Having completed the first book, he chose flashcards with pictures and letters continuing to play through related comments. Likewise, when Robert reached the predetermined criterion of 5 min, his bracelet was removed.

## 3. Results

The related graphs show the results for the conditions examined in this study. 

**Figure 1:**
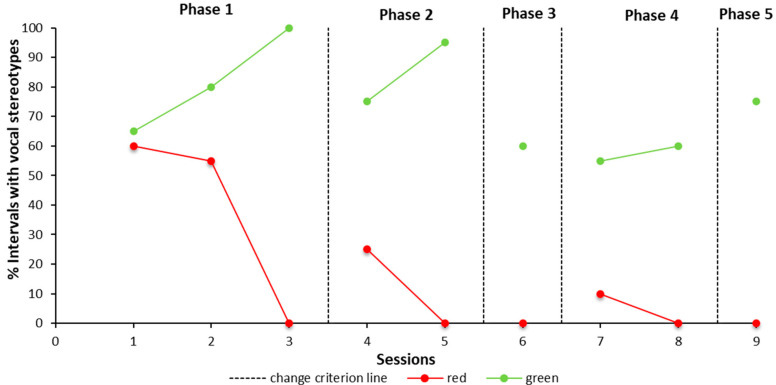
Discrimination Training, vocal stereotypies showed for both of the conditions (red card/green card) during five phases. Note: Percentage of 15-s intervals in which the child emitted the stereotyped vocal behavior. During phase 2 the red card was replaced with a red bracelet. The red lines represent the red card condition and the green lines the green card. The dotted lines (change criterion) represent the different phases where reinforcements (tangible and praise) were delivered through a variable interval schedule.

Concerning the discrimination training, at the beginning of phase 1, the child did not discriminate between the red and green stimuli. During the second session of phase 1, Robert engaged in stereotypy for the 55% of the intervals in the red bracelet condition and 80% in the green card condition. In session three, he demonstrated a clear discrimination between the two conditions (percentage of the stereotypies in red/green condition correspondingly 0/100%). Furthermore, in the other phases, discrimination between stimuli was observed. Finally, in the last phase, Robert did not emit any stereotypy in the red condition and engaged in stereotyped behaviour approximately 75% the green condition. In order to gather more information, please see Figure 1.

**Figure 2:**
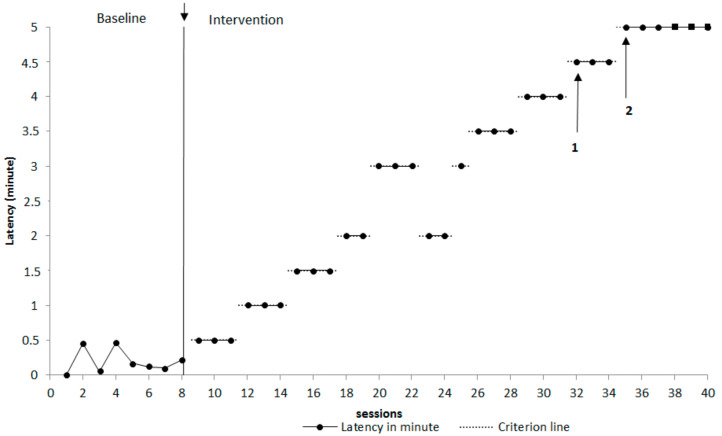
Latency in minutes to engage in stereotyped vocal behaviour during baseline sessions and interventions. Note: Arrows with numbers 1 and 2 indicate fading steps of the green card stimulus (Arrow #1 = fading sessions 32–34; Arrow #2 = no-card sessions 35–40). The arrow between the BL and intervention indicates discrimination training between the baseline and the intervention. Also, the dependent variable is the latency in minute, hence the time spent between the end of the given stimulus (“now you can play”) and the beginning of the stereotypy. The horizontal dotted lines represent the pre-established criteria in order to provide the reinforcement (access to the green card condition). The predetermined criterion is gradually increasing.

Regarding the baseline and intervention, the sessions of the baseline lasted one minute. Latency in engaging in stereotyped behaviour was of 11.7 s (average). On the other hand, the intervention was divided into sub-phases, each of which corresponds to a different reinforcement criterion. Each phase gradually approached to the final behavioural goal; the latency gradually increased. In sessions 23 and 24, a bi-directional change was observed, a return back to a previous criterion was implemented since the latency to engage in stereotypy was 2 min (as previous sessions 18–19), a useful return to a previous criterion concurred to evaluate the functional relationship between the variables, as can be seen in the Figure 2. The arrows on the graph indicate the fading steps of the green card stimulus. In sessions 32–34, the green card was faded in colour. Then, from session 35, the green card was eliminated (no card). Sessions 38–40 refer to the generalization of the intervention. In the generalization phase, sessions took place in the clinical room and the child shared play with a peer. In this phase even after the red bracelet condition, the child continued to play commenting the pictures appropriately without vocal stereotypies.

## 4. Discussion

Children diagnosed with autism spectrum disorders can display impairments in a large variety of developmental abilities, including behavioural challenges. Stereotypes including core symptoms often become for people an obstacle to academic learning, incentivizing their social stigma and limiting social interactions. However, literature reviews suggest that behavioural interventions have demonstrated efficacy in decreasing and replacing vocal stereotypies [20,41]. Moreover, recent scientific literature concerning the function of these repetitive behaviours suggest that stereotypies are maintained by non-social consequences since they generally persist in the absence of social reinforcement [20]. Vocal stereotypies maintained by automatic reinforcement are difficult to treat [21]. Some studies have used mild punishment or response interruption and redirection [22]. According to Rapp et al. [27] and O’Connor et al. [36], the results of this study indicate that stimulus discrimination training is a helpful intervention for reducing vocal stereotypy in an autistic child. In this study, we find that vocal stereotypy is under the control of the red card/green card. Moreover, the stereotyped behaviour occurs less in the presence of the red card and more in the presence of the green card, due to the inhibitory stimulus control, inducing a decrease of the stereotypies during red card condition. Furthermore, during the intervention, the green card stimulus or simply the removal of the red card, becomes a stimulus evocating automatic reinforcement, applied contingently to the absence of stereotypy in the red card condition. Actually, the access to stereotyped vocal behaviour in the green card/no card condition becomes a reinforcement in the red card condition since the child engages to decrease the vocal stereotypies. It will be interesting to study the increment of functional vocalizations of children in order to understand if a RIRD procedure [42,43], where the therapist block the self-stimulation prompting the correct response is enough to address these challenging behaviours. Future research studies could investigate the efficacy of stimulus control, comparing it with behaviour redirections. On the other hand, we believe it essential to explore the predictors of functional language development. Note that Robert showed an intermediate educational curriculum regarding language, and as a result the personal characteristic could have influenced the outcomes. As a result, it is necessary to gather more information concerning stimulus control and RIRD applications with children with different linguistic levels, especially about children following base curriculum and poor expressive language skills. Likewise, we suggest that one should conduct group analysis or multiple baselines across participants in order to address the mentioned methodology issues. Nevertheless, the current results encourage the research in this research field, demonstrating a clinical impact on autistic people and related disabilities. In fact, it has been observed that Robert, when he reached the predetermined criterion removing the red bracelet, he continued to play appropriately commenting contextually play activities. As a result, we suggest to gather more information about behaviours occurring during no card conditions and related functional comments of children.

However, the current study has other limitations. Firstly, we have noted a lack of observers’ agreement during the baseline and the intervention. The educational plan of the child was divided between the centre and home providing a one-to-one therapy. Hence, it is no easy to enrol a second observer to collect data. Another important limitation is the no-extension of the intervention to the other contexts of the daily life. Nevertheless, the use of the bracelet should encourage a generalization of treatment to public places (e.g., school, gym). Additionally, we have provided a poor definition of appropriate behaviour. It was not directly measured as well. These limitations are all aspects to be considered for the future. Regarding future studies, in our opinion measurements regarding the description and the increment of appropriate behaviour could be added. In addition, validating the effectiveness of the procedure, this study confirms that it is possible to use a stereotype to reinforce, in fact, the absence of the stereotype itself. Also, following researches could evaluate whether the results are maintained for a long time (follow-ups) or whether it is necessary to revise the training adding or removing procedural components [44].

Despite these limitations, in part related to the COVID-19 emergency, this study demonstrates how automatic behaviour can be addressed through training in discrimination and stimulus control, and how the access to stereotypy can be used as reinforcement. Finally, we would like to show to students and behavioural therapists different evidence-based strategies to address challenging behaviour in children with neuropsychiatric disorders, comprising vocal and motor stereotypies [45]. Similarly, we would suggest that any behavioural interventions should be trained by a behaviour analyst and certified therapists (generally with Master in ABA). Generally, a certified training appropriate to implement these behavioural programs should respect predefined requirements. Internationally, people can accomplish the task list concerning registered behaviour technician in order to learn contents of ABA interventions and training with behavioural analysts (https://www.bacb.com/wp-content/uploads/2020/05/RBT-2nd-Edition-Task-List_181214.pdf, accessed on 15 October 2021), although in these cases affording persistent challenging behaviours a more high-level training could be necessary (as assistant analyst). Finally, the behavioural treatments should be always supervised by a Board-Certified Behaviour Analyst (BCBA). As reminded during the method section a functional analysis assessment a tailored educational program including evidence-based behavioural interventions is necessary to guarantee the efficacy of a training about dysfunctional behaviours.

Concerning parents there are various aspects to consider. Firstly, parents of children emitted stereotypies in more contexts should receive a specific training, overall, on the implemented procedure, planning their parent inclusion. Also, parents should provide planned activities to child in order to facilitate the generalization process. Concerning the current training including stimulus control, the parents could deliver red card conditions to reduce the stereotypy, in particular during daily activities in community (parties, restaurant, playgrounds, gym, and so on) without providing the access to vocal stereotypies as reinforcement (in the current procedure during green card/no card conditions). Generally, parents of children with ASD suffer perceived problem behaviours emitted by the child, and as a result they could be encouraged to provide only punitive consequences, avoiding to furnish self-stimulations as reinforcement. All of these aspects should be considered during parent training. On the other hand, there are various positive side effects of these interventions. Commonly, problem behaviours limit daily life of families and their social relationships. Consequently, when families start a well-organized educational program including an effective parent training, it permits them to meliorate their quality of life. Likewise, stereotypy could represent a limit in classrooms either limiting learning or social interaction, increasing social stigma [10] and learning opportunities [9]. Therefore, clinical staff should select an appropriate behavioural technique in order to reduce the motor and vocal stereotypies in school settings or generalizing effective procedures implemented in structured ones. Accordingly, behavioural procedures based on stimulus control, interruption and redirection should be considered. Conversely, these interventions generally need a private cost (50 USD/hour) and many families are not available to afford it. In these cases, it could be necessary a study of cost/benefit regarding the efforts involved in planned and supervised training.

## 5. Conclusions

Children diagnosed with autism spectrum disorders are also characterized by restricted and repetitive behaviours, vocalizing, echolalia, and a-contextual speech. These are examples of stereotypies maintained by automatic positive reinforcement becoming a barrier to learning if their reinforcing value exceeds other stimuli in the environment, absorbing too many daily activities of children and their families. Undeniably, stereotyped behaviours interfere with different aspects as learning in academic setting, social skills, leisure time, and vocational and self-care time. In order to address these challenging behaviours, some behavioural interventions have been proposed in the literature including mild punishment, response interruption and redirection to appropriate vocalizations, response blocking, antecedent based interventions, reinforcement schedules, and discrimination training. Likewise, the current research suggests that stereotypy can pass under the card’s control occurring less in the presence of the red card and more when paired with the green card/no card. During the intervention, we trained a child to discriminate two conditions (red card and green card) evocating through RIRD procedure two opposed behaviours (respectively the absence and access of vocal stereotypies). Subsequently, after the discrimination training we furnished an intervention where the child watched a red card (or red bracelet) and without prompting had to maintain gradually a greater time without vocal stereotypies. In case the child respected the latency time requested, he could access to vocal stereotypies (green or no card) for the residual time of the session. Therefore, during the sessions the child increased the time passed without stereotypy, and as a result the current procedure indicates that stimulus discrimination training is a useful intervention to reduce vocal stereotypy in an autistic child. Likewise, these behavioural interventions applied by certified professionals could support therapists and families in different daily life settings as playgrounds, school, the home, and the community. Finally, a training to reduce stereotypy should be derived from individualized educational plans and appropriate functional analysis assessments combining manualized behavioural techniques in order to maximise the outcomes.

## Figures and Tables

**Table 1:** Sessions of the intervention with related changing criterion time.

Sessions (Range)	Criterion Time (Seconds)
1–8	baseline
9–11	30
12–14	60
15–17	90
18–19	120
20–22	180
23–24	120 (>stereotypies)
25–25	180
26–28	210
29–31	240
32–34	270 (fading green card)
35–40	300 (no card)

Note. The sessions lasted 5 min, the latency criterion (30 to 300 s) to access to stereotypy gradually increased. During sessions 23–24 the child needed a backstep since he reported a problem to maintain functional behaviors for a longer time. From session 39 we faded green card since the only removal of red card could evoke the automatic behaviors.

## Data Availability

In order to receive the data, the user need to write at info@unabreccianelmuro.org.
